# The Foreign Journals: Anatomy and Physiology

**Published:** 1840-07

**Authors:** 


### PART THIRD.

### Selections from the British and Foreign Journals.

### I. THE FOREIGN JOURNALS.

### ANATOMY AND PHYSIOLOGY.

*Researches on the Structure of the Cortical Layer of the Convolutions of the Brain.* By M. Baillarger. (*Abstract of the Report of MM. Ribes and Blandin.*)

The author, whose examinations have been chiefly made on the brains of maniacs under the care of M. Esquirol, considers the gray substance of the convolutions as formed of six superposed layers, differing in structure and in colour: the first passing from without inwards is white; the second gray; the third white; the fourth gray; the fifth white; and the sixth gray. This stratification may sometimes be distinguished by the naked eye on a section of the brain; but M. Baillarger has for the most part made his observations by examining by light transmitted from a lamp a thin slice of brain placed between two glasses. In this way, the gray layers easily allow the light to traverse them, while the white reflect it and remain opaque, a fact which the commissioners of the report fully confirm.

The circumstances which, according to the author, have led previous observers away from the truth are that sometimes the two deep gray layers are atrophied, so that the two corresponding white layers confounded together cannot be distinguished from the medullary substance of the convolutions, and the whole of the cortical part here seems formed of but two layers; and that in other cases the middle gray layer is so thin that the two adjacent white layers are united, and the cortical substance looks all gray except a middle band of white.

The gray substance is not merely apposed upon the white, as Reil imagined, but the latter penetrates into the interior of the former. The fibres by which this union is effected are conical with their points directed towards the surface of the brain. They are very numerous and vertical towards the summit of the convolutions, but are less abundant and oblique on the sides, and lastly are very few in number, and are directed transversely towards the bottoms of the anfractuosities; some terminate in the most internal gray layer; others penetrate more deeply to the superficial gray layers, becoming thin where they pass through gray layers, and swelling when they arrive at the white ones. The white layers are in great measure formed by the above-mentioned swellings of fibrils; but they seem besides to possess fibrils of their own, which are parallel to the surface of the brain; at least, fibres of this kind have been found in dogs and rabbits.

The most external white layer of the cortical substance, which is immediately subjacent to the pia mater, is often torn off with that membrane when the part beneath has been a little inflamed. It may be separated from a healthy brain by placing a dry cloth for a few instants on the surface of the convolutions after the membranes are taken off; by raising the cloth the layer in question is removed with it. It seems of the same nature as the central white substance, and it is remarkable that it often preserves its white colour in certain lunatics even when the subjacent gray substance is vividly injected.

The yellow layer which Gennari, Vicq-d'Azyr, and others ascribe as placed between the cortical and medullary parts of the brain, is the result (according to M. Baillarger) of an atrophy and discoloration of the deepest gray layer.

Contrary also to the opinion of Reil and Tiedemann, M. Baillarger has detected the gray layer of the convolutions from the age of four months of intra-uterine life, recognizing it, as in the adult, by its alternately gray and white stripes.

The discoveries of M. Baillarger (it is added by the commission) call the attention of pathologists to the different layers of the gray substance, which in certain cases, as in the insane, may be separately affected. The expectations, moreover, that their investigation may lead to important facts are not merely theoretical; they have been already confirmed by practical results. M. Foville long since pointed out the separate hypertrophy of the external white layer, and the inflammation of the gray layer beneath it in some cases of insanity, and his observations have been repeated by MM. Parchappe and Baillarger. Nor is it in a pathological point of view only that the importance of this stratification of the cortical substance is to be considered; it seems to be at least as important in physiology, for it appears connected with the functional perfection of the brain: it is well developed only in mammalia and especially in man, and it is never met with in birds, reptiles, or fishes, in which it seems to take refuge in the optic lobes, which, in this respect as in many others, form a kind of substitute for the cerebral hemispheres.

*Bulletin de l'Acad. Roy. de Médecine. Fevr.* 15, 1840.

---

*On a Peculiar Monstrosity.* By M. FAESEBECK, of Brunswick.

IN a letter to Professor Müller on the arrangement of the cervical portion of the sympathetic nerve, the author mentions the case of a male child, twelve weeks old, and still living in his town, with a very singular abnormal formation. Above the umbilicus it is well made; but just to the left and a little above the level of that part there grows out of it a semitendinous cylindrical body, about an inch and a half thick and an inch long, on which there is a second pelvis with two extremities and sexual organs. These parts are placed transversely across the child's abdomen. The remarkable circumstance is that the child makes water through both penises; but that which flows from the second penis is only a turbid fluid like urine, and sometimes white and milky; and this last character is observed only soon after the child has been sucking.

[Probably the digestive canal of the perfect child divides at a short distance from the stomach, and a portion passes into the appended body, when it soon terminates in a cloaca, of which the urethra is one orifice.]

*Müller's Archiv.* 1840. Heft i.

---

*On the Structure of the Macula Lutea (Foramen of Soemmering) of the Human Eye.* By Dr. GRUBE, of Konigsberg.

THE author had previously made many observations on the structure of this part, but their results had been unsatisfactory in consequence of the length of time that had elapsed after death before the eye was removed. The examination here described is that of an eye taken from a body a few hours after an accidental rupture of the spleen.

The retina adhered so firmly to the vitreous humour that the greater part of the latter could be removed only by carefully cutting with scissors. With the naked eye it was at once easily discerned that the level of the macula lutea was not a little raised above the surface of the retina. On placing it and the part around it under a microscope magnifying 300 times, and compressing it but slightly, the macula lutea presented exactly the appearance of shagreen. Longish-round corpuscles, which were smaller the nearer they were to the centre, and there not more than one fourth or one fifth of the size of the medullary corpuscles on the surface of the rest of the retina, were arranged close together and with great regularity, like rays passing from the centre to the circumference of the spot. Towards the circumference they became larger, and gradually merged into the form and size of the corpuscles of the rest of the retina. The circumference, however, did not form a regular circle, but the small medullary bodies radiated beyond it at some parts, to different distances from its outline.

*Müller's Archiv.* Heft i. 1840.

---

*Notice of a New Monstrosity, a portion of a Fœtus living upon the Testicle.* By M. VELPEAU.

THE remarkable case here detailed is that of a tumour removed from the scrotum of a young man æt. 27. He had always had good health. The tumour, which was the size of a fist, had existed from birth, but was at first small and deemed unimportant: it had continued to grow slowly up to the time that the patient applied at La Charité. Various opinions were formed as to its nature; but M. Velpeau—considering that it was congenital and at first scarcely observed, that it had never given any pain and had excited no morbid process, that it was insensible to pressure, and might be cut or pricked or pierced through and through without giving the least pain, and observing the appearance of the skin that covered it which was quite different from that of the scrotum, as well as its elasticity and hardness internally, and a tuft of hair which protruded from a kind of ulcer at its posterior part, and a reddish tubercle at the bottom of another aperture anteriorly from which glairy and grumous matter had sometimes been discharged,—hit upon the idea that it was a fœtal tumour, a product of conception. He therefore determined to cut it off, to perform *a Cæsarian operation upon the man.*

On examining the tumour afterwards he found in it almost all the elements of a human body. Its exterior was evidently cutaneous, and the greater part of its substance was a mixture of lamellæ and fibres like cellular, adipose, muscular and fibrous tissues. In its interior there were two cysts filled with substance like albumen or the vitreous humour; another cyst as large as a partridge's egg containing a greenish semi-liquid matter like meconium; and a fourth containing a dirty yellow grumous mass surrounded by hairs; the mass consisted of sebaceous matter and scales of epidermis; the hairs had no bulbs. The tuft of hair that protruded externally, proceeded from the cyst that contained the meconium-like substance, and they gave the opening into it somewhat of the appearance of an anus. Lastly, in the midst of all these, numerous perfectly organized portions of a skeleton were found, consisting of bones more or less closely resembling the clavicle, scapula, humerus, sphenoid, sacrum, and portions of vertebræ, and others whose names could not be determined.

M. Velpeau remarks justly on the peculiarity of this case, that it is distinguished from all others of monstrosity by inclusion, by the second fœtus not existing as a foreign body in the other, but having a separate and independent existence and power of growth within itself. The tumour had its own colour and consistence, and a sensibility entirely independent of the individual to whom it was attached; the man himself several times pierced it with a knife without feeling the least pain, and yet all the wounds that were made in it bled and inflamed and cicatrized like those of any other part of the body. There was nothing in it that indicated the least morbid condition; all the substances found in it gave the idea of normal tissues or products, and not a drop of pus, nor any caries or necrosis of bone, nor any altered cartilage or the least fungous growth could be discovered.

The suggestions which M. Velpeau throws out for the explanation of this singular production are that it is a portion of a fœtus which in intra-uterine life became attached to the scrotum, and remained there like a graft; or that it is the remains of a fœtus which passed into the abdomen of another, and then having descended into the tunica vaginalis, at last gradually made its way through the envelopes of the scrotum; or that it is a separate creation, the product of the testicle alone.

*Gazette Médicale. Fevr.* 15, 1840.

---

*On the Lymphatic Hearts of the Tortoises.* By Professor Müller.

The tortoises were the only order of amphibia in which the lymphatic hearts had not been found, although the lymphatic vessels had been examined in them more frequently than in any others of the class. The author gives the following account of them in the Turtle (chelonia mydas), in which of all the amphibia they are most easily found.

The two hearts lie under the posterior part of the great median plate of the shell. If the median line of this plate be divided into three equal parts, and through the points of division lines be drawn transversely across the plate at right angles to the median line, the second of these transverse lines which separates the second and third portions from one another indicates the position of the two hearts. If this transverse line be now divided into three equal parts, the two points of division will exactly mark their respective situations. To expose them a quadrilateral piece including these points must be cut out of the shell and carefully separated. A lymphatic heart lies on each side, just behind the upper extremity of the ilium; its lower wall rests upon the origins of the musc. semi-tendinosus and semi-membranosus, and its outer side is bounded by the inner edge of the origin of the biceps.

The heart is of an irregularly rounded form, rather flattened above, and, in a turtle of 140 lbs. weight, was an inch in diameter. On its outer side it received a bundle of very large lymphatic vessels which brought to it the lymph of the posterior extremities; and at its hinder part others which brought the lymph from the posterior portion of the abdominal walls.

Both hearts contracted regularly and powerfully three or four times in a minute, sometimes but not always synchronously. When one of them was cut out a quantity of lymph flowed from it at each contraction. After they had been examined for several hours, the turtle's head was cut off, but their movements still continued; and next day, when all the abdominal viscera were removed and the shell cut across, they still went on contracting, though more weakly than before. If the hind limbs attached to the posterior part of the divided turtle were now irritated, the common reflex motions of the animal muscles took place, and the lymphatic heart of the same side contracted.

The internal surface of these hearts is in general smooth, but the apertures leading into them from lymphatics are variously arranged. At all these entrances there are valves so placed that the heart can never drive the lymph backwards when it contracts. On the inner side of the heart there is a vein which is formed of the union of several smaller veins from the posterior part of the body, and which receives the very short efferent lymphatic vessels from the anterior and inner part of the heart. The vein then passes under the union of the pelvis with the vertebral column, and unites with several other veins from the muscles of the thigh into a considerable trunk, which becomes the vena renalis advehens and is connected with the umbilical vein.

The author's observations on the lymphatic hearts of crocodiles agree with those of Panizza. He has many times sought in vain for similar organs in fish, but he has no doubt of their existence in them as well as in amphibia.

*Müller's Archiv.* 1840. Heft i.

*On the Microscopic Characters of the Menstrual Blood.* By Dr. BUROW, of Königsberg.

DR. BUROW examined twelve ounces of menstrual blood which had been retained in the uterus by an imperforate hymen. The blood was of a dirty reddish-brown colour, of the consistence of syrup, very adhesive, and perfectly destitute of smell It abounded in albumen, and was very little susceptible of putrefaction.

When looked at under the microscope almost all the blood-globules were seen to have lost their regular form and to resemble those granules which may be observed in pus which has been for a long time exposed to the air, or retained within the cavity of an abscess. These blood-globules were suspended in a transparent fluid. On stirring the blood for a considerable time no change perceptible to the eye was produced, but under the microscope a great number of delicate transparent lamellæ were seen floating in the serum, and these Dr. Burow regards as portions of fibrine, which substance is sparingly present in menstrual blood.

*Muller's Archiv.* No. vii. 1840.

---

*Novel Researches concerning the Action of Madder on the Bones.* By M. FLOURENS.

THE novelties contained in M. Flourens' papers are the proofs of the remarkable rapidity with which nutrition is effected in the bones of young animals. Among the preparations exhibited to the Academy of Sciences was the skeleton of a pigeon between two and three weeks old, which had been fed on madder mixed with its food for only two days, and had taken at most three grammes of alizarine (an alcoholic extract of madder); its bones were deep red. Another preparation was the skeleton of a pigeon which had fed on alizarine for only one day and whose bones had all a distinctly red hue. A third was the skeleton of a young pigeon which was killed twenty-four hours after eating the only meal in which madder had been mixed with its food; all its bones were vividly red A fourth was that of a pigeon which five hours after having taken food containing six grammes of madder was killed ; the bones, though less coloured than those in the preceding cases, were yet very red : so that it it appeared that for madder to pass through all the processes preliminary to nutrition, and to penetrate into and be incorporated with the bones, a period of five hours only was necessary.

M. Flourens confirms the results of previous experiments, that the madder has little or no influence in colouring the bones of old animals ; and he states that the dyeing is effected with different degrees of rapidity by different kinds of madder. That from Avignon always required a larger dose and a longer time to produce a given effect than that from Alsace did.

*Gazette Médicale. Mars* 14, 1840.

---

*On Dr. Marshall Hall's Doctrine of the non-participation of Sensation in the (so called) Reflex Motions.* By F. NASSE, M.D. of Bonn.

THE object of the author in this essay is to show the supposed error of that opinion which was hinted at by Sir Gilbert Blane, but first assumed as proved by Dr. Marshall Hall, that neither sensation nor the psychical principle in general have any influence in the excitement of reflex motions through the medium of the spinal cord in beheaded animals. He enters into a rather long statement to show that many authors before Marshall Hall were well acquainted with the phenomena of reflex motion, and with their connexion with the brain and spinal cord, though they had no idea of excluding the mind from any influence in them. He quotes especially from Whytt, Legallois, Hales, and

Soemmering, and in no indirect terms accuses M. Hall of suppressing their opinions and some others which were adverse to the correctness and originality of his own.

To prove the correspondence between the motions of beheaded and those of uninjured animals the author maintains,—1st, that motions are observed in the former which are clearly indicative of purpose and intention; 2d, that these motions are performed not merely after external stimuli, but from internal impulse; 3d, that when they are excited from without they ensue most easily after stimuli, which would have excited the same or similar motions in perfect animals: and lastly, that they take place most easily after stimulating those parts which in uninjured animals are peculiarly sensitive.

The facts by which these four propositions are supported are drawn from various sources, and especially from the experiments of M. Hall and Grainger on beheaded animals. Most of them are generally known; and the evidence which they afford of the similarity of psychical condition in the beheaded and the entire animal in respect of perception and of capability of reacting upon impressions, *with purpose and intent,* seems to Dr. Nasse incontrovertible.

The next chief section is occupied with the consideration of the circumstances which indicate a difference between the psychical conditions in the two states. The most remarkable of these are: 1st, That an entire animal can be excited to motion by an impression of any of its senses, but a beheaded one only by those which act on the sense of feeling. 2d, That injuries of the parts that lie deeply beneath the skin produce few or no signs of pain in beheaded animals. 3d, That while an animal with its spinal cord divided moves the parts above the division whenever they are irritated, yet the parts below it are quite motionless, even when those above it are moving with the greatest activity—facts which are observed in men as well as in animals.

These objections the author opposes by showing: 1st, That when all the sensitive nerves but those of common sensation are removed from an animal it is only natural that motions should be excitable only by such impressions as in the perfect animal would act on the sense of touch. 2d, That the absence of signs of pain is not so distinct as it is commonly supposed to be, and that if animals do not move when deeply-seated parts are irritated they may yet have sensation of their skin; because the perception of pain and common sensation probably depend on different psychomatic functions, as insects, the surface of whose body has an exquisite sensibility yet will allow the cutting off of their antennæ or limbs without any indication of having suffered harm. 3d, That although in man when the spinal cord has been divided there is no longer any sensation in parts supplied from the lower portion, yet this may depend in some measure on the injury which that portion has at the same time undergone. In a case by Dessault, in which the division though complete was unattended by injury of the rest of the cord, voluntary motions of the parts supplied from below the division were distinctly observed: and in another by Ollivier when the separation from disease was found complete after death, there was distinct evidence of voluntary motion in the lower parts. In the lower animals, however, there is no doubt of intended motions being performed for definite purposes by the limbs which are entirely separated from the influence of the brain; they are constantly observed in beheaded frogs, insects, &c., and even in dogs and birds whose brains have been removed.

If therefore, the author concludes, we put together the results of these investigations the balance is not in favour of the doctrine which assumes that there is no direct psychical influence of the spinal cord. That which, before it was anatomically and psychologically considered, appeared to have but little foundation is proved by the undeniable intention of the movements which take place without the influence of the brain, and by the occurrence of such motions without any external stimulus, as well as by the proofs of sensibility continuing in the skin without the participation of the brain—to be an untenable opinion.

The rest of the paper scarcely admits of analysis. It treats chiefly of the amount and kind of psychical power which the spinal cord may be considered to possess ; and is concluded by a more detailed consideration of the nature of some of the actions which are performed under its influence when uncontrolled by that of the brain.

[We have thought it due to Dr. Nasse to present this statement of his views to our readers; but we by no means concur in their justice. The objections which he brings against Dr. M. Hall's views are in the main the same which were formerly adduced by Volkmann, and which we noticed at the time (Vol.VI. p. 211-17.) As to the objection founded on the *adaptive* character of the movements in question, we need say nothing in addition to what we then remarked (p. 213.) And in regard to other points our opinions have been fully confirmed by pathological evidence of the most satisfactory description in the recent paper by Dr. Budd, on the pathology of the spinal cord, in the Medico-Chirurgical Transactions. Now that the attention of practitioners is directed to the subject, we have no doubt that in a few years a body of cases of reflex action without sensation or volition will be collected quite sufficient to determine the question in dispute.]

*Untersuchungen zur Physiologie und Pathologie.* 1839.

---

### *Microscopical and Experimental researches on Softening of the Brain.* By Professor GLUGE.

[We have pleasure in transferring to our pages the general results of the first attempt (as we believe) made to elucidate the nature of certain morbid changes of the brain by a practised micrographer, well acquainted with the normal microscopic structure of that viscus.]

1. Softening of the brain may be either partial or general. 2. The softened substance may retain its primitive colour, but this is the rarest form of the disease; under these circumstances there may be pus present in the softened fragments. The pus is often visible with the naked eye; more commonly the microscope is required for its detection: the nervous canals (or tubuli)* are mingled in fragments with the globules of pus. No morbid product is observable in the softened brain, but the nervous canals are, under the microscope, found to have lost their natural soundness, or to be reduced to fragments : this I have sometimes observed in the softened brain of typhoid fever; it is utterly unconnected with inflammation. I have not observed any distinct example of white softening accompanied with symptoms of paralysis, and independent of inflammation, but admit that some published facts render the reality of its occurrence very probable. 3. The colour is ordinarily changed to pink, yellow, or gray. The central substance presents, in the majority of cases of softening, and *invariably when there is no apoplectic effusion*, the peculiar *compound globules* already described,† which we have shown to be a product of inflammation, and susceptible of being produced in the various tissues of animals. These globules form in the capillary vessels, and obstruct the circulation: exhibition of the serum through the walls of the vessels follows, and causes *mechanical maceration* of the brain. The globules of the blood, as is well known, promptly communicate their colouring matter to the serum, hence the altered colour of the softened cerebral substance. Subsequently the vessels give way, and the compound globules mingle with the fragments of macerated brain. The nervous canals (or

* For the meaning of this term we must refer the reader to the account given in our Sixth Volume (p. 393, et seq.) of the discoveries of Ehrenberg, Treviranus, and others upon the normal intimate structure of the nervous tissues.

† "In one of the earliest stages of inflammation the blood globules lose their colour and size, run together into a mass to the number of thirty or forty, obstruct the interior of the vessels, and put a stop to the circulation; the accumulated masses are of blackish colour, and, for shortness sake, may be called *compound globules.* They are 1·30 of a millimeter and more in diameter."

tubuli) are only to be found in fragments and often lose their soundness. 4. The softening may take place round an apoplectic effusion ; in this case I have observed two changes. In one of these, and more especially in apoplexy of old standing, compound globules are commonly found as the result of inflammation excited by the mechanical agency of the effused blood. In other cases imbibition of blood into the cerebral substance alone takes place, and produces maceration and softening without exciting inflammation Softening may be very readily produced experimentally, by introducing a large pin into the brain of a rabbit; an effusion of blood gradually takes place, and consecutively softening of the cerebral substance surrounding the accumulation. It is only when the effused blood sojourns longer in the brain that inflammation may be established. The experiments which I have made do not in the least support the opinion held by some (e. g. Rochoux) that softening is a regular forerunner of apoplexy. Besides, why should not rupture of the capillary vessels from the impetus of the blood, or from some alteration of their structure, quite as plausibly explain the occurrence of hemorrhage as preexisting softening?

The following results may be easily verified by any one who possesses a microscope and even a superficial acquaintance with the structure of the brain:— 1. Pus is present in many examples of white softening. 2. In coloured softening without effusion of blood there is formation of compound globules. 3. Coloured softening with effusion of blood may present the preceding productions, or simply mechanical imbibition of sanguinolent serum ; as is commonly the case in recent apoplexy. The greater number of cases of softening hitherto observed by pathologists belong undoubtedly to the second class, and are consequently results of a degree of inflammation, the nature of which had, until these researches were undertaken, eluded discovery.

*Archives de la Méd. Belge. Janv.* 1840.*

---

### *Formation of Muscular Fibres in a dilated renal Pelvis and Ureter.*
By Dr. Tourtual, of Münster.

The subject of this interesting paper was a scrofulous young man, twenty years old, who had suffered for many years from tuberculous disease of the urinary organs. At the examination of his body both kidneys were found twice as large as usual, and the ureters very much dilated. The urethra from the bladder to within three inches of its orifice was so dilated that it looked like a somewhat narrowed continuation of the bladder, and no limit could be seen between the prostatic and membranous portions and the bulb, the vesical orifice being marked merely by a transverse fold across the posterior wall of the bladder, and the prostate being so distended that it was scarcely discernible. All this dilated portion presented an ulcerated surface, which extended upwards into the thinned and condensed corpus cavernosum, the corpus spongiosum urethræ having been almost entirely removed. The lining of the bladder was almost all ulcerated and covered with pus, and its muscular tissue was excessively hypertrophied. Both the ureters were shortened and remarkably dilated, being nearly an inch in diameter by the bladder. The left kidney was hard and tuberous, and made up chiefly of several large sacculi like cavities in the lungs. The walls of the corresponding ureter were thickened and hard and condensed by the effusion of lymph into its cellular and submucous tissues.

The more remarkable changes were found in the right kidney. Its pelvis was distended into a cavity capable of holding three ounces of water. The walls of the ureter were thicker than usual, but they were not morbidly thickened, nor did they exhibit any sign of inflammation having existed. The internal membrane was thrown into prominent folds which lay across its canal, and, when it

* The first number of a new Brussels journal which promises to prove an important acquisition to periodical literature.

was opened, gave it the aspect of a vesicula seminalis. This lining membrane was easily separated; and on the inner surface of the surrounding cellular tissue muscular tissue consisting of yellowish red circular fibres was evidently developed. The muscular fibres were plainest at the most dilated parts; and at the pelvis they formed a plexus like the fasciculi of the bladder, but finer, the fibres crossing each other at very oblique angles. In the pelvis, also, on the exterior of these circular fibres, fasciculi of longitudinal fibres were visible both anteriorly and posteriorly which expanded and were lost upon its walls. In like manner circular fibres were discerned at the lowest part of the ureter next the bladder; and longitudinal fibres upon the dilated part of the urethra which were covered anteriorly by the bulbo-cavernosus, and posteriorly were continued over the prostatic portion to the detrusor urinæ, and which could not therefore be regarded as belonging to the bulbo-cavernosus.

An examination of all these fibres left no doubt of their being truly muscular. Their yellowish red colour, their remarkable size, and their regular uninterrupted arrangement in rings along the whole ureter, all supported this opinion. They were moreover soft, slightly extensile, but scarcely elastic, and they tore across when they were stretched. The fibres were roundish, and connected together by cellular tissue; they did not pass into each other but were parallel to one another. Their situation also between a mucous and a cellular membrane, with each of which they were connected by cellular tissue,—their arrangement in an internal circular, and an external, though incomplete, longitudinal layer like the muscles of the whole digestive canal—the similarity of the dilated ureter itself to an intestine—the coincident hypertrophy of the muscular coat of the bladder and of the bulbo-cavernosus—and the evident uses that a development of muscles in these parts would have—are further grounds for the belief that these were actually muscular fibres.

A chemical examination was made of a portion of them from each part. On being placed in concentrated acetic acid, all the pieces became pale, transparent, and jelly-like, and on the following morning were dissolved into a whitish cloud at the bottoms of the glasses. On adding some drops of a solution of ferrocyanate of potash to these filtered solutions, they became turbid, exhibited some yellow flocculi, and by the next day had thrown down an abundant yellow-green precipitate which, on exposure to the air, became dark green.

The result of this analysis being confirmatory of the view already adopted, the fibres were next subjected to microscopic examination. With a power of 200 times they appeared of a clear greenish-yellow colour, and as it were clouded by their cellular sheath; transverse striæ could not be discerned upon them; but at their extremities, where the cellular sheath had retracted, the primary filaments were discerned in the form of constricted threads like strings of beads, which were straight or but slightly curved and parallel, and seemed composed of rather dark oval corpuscles. The transverse diameter of these filaments was about one fourth of that of a blood-globule.

These fibres were lastly compared beneath the microscope with those of all the other tissues with which it was possible they might be confounded, but they were found distinctly different from each, and there could be no reasonable doubt of their genuine muscularity. The author observes that this is the first case in which muscular fibres have been proved as the product of disease in a part where none previously existed; but he correctly adds that these are not to be regarded as the immediate result of a morbid process but as the effects of an exalted power of development comparable with hypertrophy. As far as previous examinations had gone, the human ureter possessed only a cellular and probably contractile tissue; but Meyer had shown that in the horse it is distinctly muscular; the present investigations prove that in man also it may, under peculiar circumstances, become muscular, its tissue passing through the transition from cellular to muscular, of which traces are to be observed in some other parts of the body.

*Müller's Archiv.* Heft ii. 1840.

